# Gene Expression Changes for Antioxidants Pathways in the Mouse Cochlea: Relations to Age-related Hearing Deficits

**DOI:** 10.1371/journal.pone.0090279

**Published:** 2014-02-28

**Authors:** Sherif F. Tadros, Mary D'Souza, Xiaoxia Zhu, Robert D. Frisina

**Affiliations:** 1 International Center for Hearing & Speech Research, National Technical Institute for the Deaf, Rochester Institute of Technology, Rochester, New York, United States of America; 2 Otolaryngology Dept., University of Rochester Medical School, Rochester, New York, United States of America; 3 Depts. Chemical & Biomedical Engineering, Communication Sciences & Disorders, and Global Center for Hearing & Speech Research, University of South Florida, Tampa, Florida, United States of America; Instituto de Biociencias - Universidade de São Paulo, Brazil

## Abstract

Age-related hearing loss – presbycusis – is the number one neurodegenerative disorder and top communication deficit of our aged population. Like many aging disorders of the nervous system, damage from free radicals linked to production of reactive oxygen and/or nitrogen species (ROS and RNS, respectively) may play key roles in disease progression. The efficacy of the antioxidant systems, e.g., glutathione and thioredoxin, is an important factor in pathophysiology of the aging nervous system. In this investigation, relations between the expression of antioxidant-related genes in the auditory portion of the inner ear – cochlea, and age-related hearing loss was explored for CBA/CaJ mice. Forty mice were classified into four groups according to age and degree of hearing loss. Cochlear mRNA samples were collected and cDNA generated. Using Affymetrix® GeneChip, the expressions of 56 antioxidant-related gene probes were analyzed to estimate the differences in gene expression between the four subject groups. The expression of Glutathione peroxidase 6, Gpx6; Thioredoxin reductase 1, Txnrd1; Isocitrate dehydrogenase 1, Idh1; and Heat shock protein 1, Hspb1; were significantly different, or showed large fold-change differences between subject groups. The Gpx6, Txnrd1 and Hspb1 gene expression changes were validated using qPCR. The Gpx6 gene was upregulated while the Txnrd1 gene was downregulated with age/hearing loss. The Hspb1 gene was found to be downregulated in middle-aged animals as well as those with mild presbycusis, whereas it was upregulated in those with severe presbycusis. These results facilitate development of future interventions to predict, prevent or slow down the progression of presbycusis.

## Introduction

Oxidative stress has a definite role in cell aging. The multifactorial approach of explaining the processes of aging in the ear and brain results in different theories. Some of these theories explain aging as an evolutionary effect (e.g. mutation accumulation theory, particularly for the mitochondrial genome), others explain it as a molecular effect (e.g., gene regulation theory), a cellular effect (e.g., telomere length theory), or a systemic effect (e.g., neuroendocrine and immunological theories).

One of the main cellular theories of aging is the free radical theory. According to this, aging is the result of accumulative effects of oxidative insults throughout the life span [1] caused by reactive oxygen and/or nitrogen species (ROS and RNS, respectively) [2]–[5]. The main source of ROS in the cell is the mitochondrial leakage of electrons, followed by production of superoxides, and hydrogen peroxide (H_2_O_2_) [6]–[7]. The lack of ability to scavenge all of these oxidants from the cell leads to DNA, lipids and protein damage [8]–[11]. Lipid damage, in turn, can lead to calcium (Ca++) influx and pathological changes in the cells [12]–[13]. DNA damage can be more permanent, cumulative and have more devastating consequences than lipid or protein damage. The fact that mitochondria continue to replicate, and that the mitochondrial genome almost entirely codes for proteins, makes mitochondrial DNA (mtDNA) oxidative damage very important and potentially dangerous to cellular health, even relative to nuclear DNA damage [1], [14]–[15].

Thiol-reducing systems, glutathione (GSH) and thioredoxin (Trx) are two major cellular antioxidant systems that play an important role in maintaining the proper intracellular redox concentration. Other redox compounds, such as nicotinamide adenine dinucleotides (NADPH, NADH), ascorbic acid (Vitamin C), tocopherols (Vitamin E), lipoic acid, and ubiquinone (Q10), in addition to antioxidant enzymes like dimutate superoxide (SOD-1 and SOD-2), catalases and peroxidases also play roles in cell protection [16]. The balance between these systems and ROS/RNS production is vital for cell survival. Decreases in levels of antioxidants along with overwhelming exposure to oxidative stress can accelerate the process of cellular age-related damage [17]–[19]. Due to its high intracellular concentration, *GSH* is thought to be largely responsible for antioxidant protective effects inside cells and organelles. In addition to its action as a major antioxidant defense, Trx may play an important role in the redox regulation of protein thiols involved in signal transduction, gene regulation and cell growth [16], [20]–[21].

Age-related hearing loss is the number one neurodegenerative disorder and top communication deficit of our growing aged population; and along with arthritis and cardiovascular disease, one of the top three chronic medical conditions of the elderly. The CBA mouse strain has been one of the most useful in terms of understanding the behavioral, neural and molecular bases of age-related hearing loss at both sensory end-organ (inner ear: cochlear) and brain (central auditory system) levels [22]–[25]. In the present study, age- and function-related changes in antioxidant-related cochlear gene expression in different age groups of CBA mice were investigated. Utilizing gene microarrays, candidate genes that may participate in presbycusis – age-related hearing loss - at the cochlear level were identified.

## Methods

The present investigation utilized methodologies similar to Tadros, Frisina and colleagues [26]–[28].

### Animal Model

CBA/CaJ mice were bred in-house and housed according to institutional protocol, with original breeding pairs obtained from Jackson Laboratories. All animals had similar environmental and non-ototoxic history. The CBA mouse is a model organism for human presbycusis because it loses its hearing progressively over its lifespan. The young adult group was used as the baseline group for gene expression data analyses (e.g., calculation of fold changes). Functional hearing measurements were obtained prior to sacrifice similar to our previous investigations of the biological bases of presbycusis [29]–[31]. Before data acquisition, individual mice were microscopically examined for evidence of external ear canal and middle ear obstruction. Mice with clearly visualized, healthy tympanic membranes were included. This study was carried out in strict accordance with the recommendations in the Guide for the Care and Use of Laboratory Animals of the National Institutes of Health, and all procedures were fully approved by the University of Rochester Vivarium Committee on Animal Resources.

### Functional Hearing Assessment

#### Distortion Product Otoacoustic Emissions (DPOAEs)

Ipsilateral acoustic stimulation and simultaneous measurement of DPOAEs were accomplished with the Tucker Davis Tech. (TDT) BioSig III system. Stimuli were digitally synthesized at 200 kHz using SigGen software applications with the ratio of frequency 2 (F2) to frequency 1 (F1) constant at 1.25; L1 was equal to 65 dB sound pressure level (SPL) and L2 was equal to 50 dB SPL as calibrated in a 0.1-mL coupler simulating the mouse ear canal. After synthesis, F1, F2, were each passed through an RP2.1 D/A converter to PA5 programmable attenuators. Following attenuation, the signals went to ED1 speaker drivers which fed into the EC1 electrostatic loudspeakers coupled to the ear canal through short, flexible tubes with rigid plastic tapering tips. For DPOAE measurements, resultant ear canal sound pressure was recorded with an ER10B+ low-noise microphone and probe (Etymotic) housed in the same coupler as the F1 and F2 speakers. The output of the ER10B+ amplifier was put into an MA3 microphone amplifier, whose output went to an RP2.1 A/D converter for sampling at 200 kHz. A fast Fourier transform (FFT) was performed with TDT BioSig software on the resultant waveform. The magnitude of F1, F2, the 2f1-f2 distortion product, and the noise floor of the frequency bins surrounding the 2f1-f2 components were measured from the FFT. The procedure was repeated for geometric mean frequencies ranging from 5.6 to 44.8 kHz (eight frequencies per octave) to adequately assess the neuroethologically functional range of mouse hearing.

Mice were anesthetized with a mixture of ketamine and xylazine (120 and 10 mg/kg body weight, respectively) by intraperitoneal injection before all experimental sessions. All recording sessions were completed in a soundproof acoustic chamber (lined with Sonex) with body temperature maintained with a heating pad. Before recording, the operating microscope (Zeiss) was used to place the stimulus probe and microphone in the test ear, and the coupler for the speaker for the contralateral noise source was placed into the opposite ear canal. Both couplers were placed close to the tympanic membrane. The recording session duration was limited by depth of anesthesia, and lasted approximately 1 hour per animal.

#### Auditory Brainstem Responses (ABRs)

During this procedure, 5.0 mg/10.0 gm body weight general anesthetic, Avertin (Tribromoethanol, delivered IP), was used to anaesthetize the mice. Normal body temperature was maintained at 38°C with a Servo heating pad. Auditory brainstem responses were measured in response to tone pips of 3, 6, 12, 24, 32, and 48 kHz presented at a rate of 11 bursts/sec. Auditory brainstem responses were recorded with subcutaneous platinum needle electrodes placed at the vertex (noninverting input), right-side mastoid prominence (inverted input), and tail (indifferent site). Electroencephalographic (EEG) activity was differentially amplified (50 or 100 X) (Grass Model P511 EEG amplifier), then put into an analogue-to-digital converter (AD1, TDT) and digitized at 50 kHz. Each averaged response was based on 300–500 stimulus repetitions recorded over 10-millisec epochs. Contamination by muscle and cardiac activities was prevented by rejecting data epochs in which the single-trace electroencephalogram contained peak-to-peak amplitudes exceeding 50 µV. The ABR was recorded in a small sound-attenuating chamber.

### Sample Isolation

Upon completion of the physiological recording sessions, the mice were sacrificed by cervical dislocation. The brains were immediately dissected using a Zeiss stereomicroscope and placed in ice-cold saline. The soft tissues of forty CBA mice cochleae were dissected. The two cochleae of each mouse were pooled together to form one sample per mouse. All samples were placed in cold Trizol (Invitrogen, CA) and stored at −80°C for gene microarray and real time PCR processing.

### Microarray gene expression processing

#### Gene Chip

One Affymetrix M430A high-density oligonucleotide array set (A) (Affymetrix Inc., Santa Clara, CA) was used for each cochlea sample. Each array contains 22,600 probe sets analyzing the expression of over 14,000 mouse genes. Eleven pairs of 25-mer oligonucleotides that span the coding region of the genes represent each gene. Each probe pair consists of a perfect match sequence that is complementary to the mRNA target and a mismatch sequence that has a single base pair mutation in a region critical for target hybridization; this sequence serves as a control for non-specific hybridization. Sequences used in the design of the array were selected from GenBank, dbEST, and RefSeq.

### Sample Preparation

#### RNA Extraction

These procedures are described in detail in our previous microarray articles [26]–[28].

#### cDNA Synthesis

For gene array analysis, cDNA synthesis was performed with 20 µg of total RNA using the Superscript Choice cDNA Synthesis Kit (Invitrogen). For qPCR, nuGen cDNA reagents kit was used to generate a high fidelity cDNA, which was modified at the 3′ end to contain an initiation site for T7 RNA polymerase. Detailed protocol is found in www.nugeninc.com.

#### *In-vitro* Transcription (IVT) and Fragmentation

Clean up of double-stranded cDNA was done according to the Affymetrix GeneChip Expression analysis protocol. Synthesis of Biotin-labeled cRNA was performed by adding 1 µg of cDNA to 10X IVT labeling buffer, IVT labeling NTP mix, IVT labeling enzyme mix and RNase-free water, then incubated at 37°C for 16 hours. The Biotin-labeled cRNA was cleaned up according to the Affymetrix GeneChip expression analysis protocol and a 20 µg of full-length cRNA from each sample was fragmented by adding 5X fragmentation buffer and RNase-free water, followed by incubation at 94°C for 35 min. The standard fragmentation procedure produces a distribution of RNA fragment sizes from approximately 35 to 200 bases. After the fragmentation, cDNA, full-length cRNA and fragmented cRNA were analyzed by electrophoresis using the Agilent Bioanalyzer 2100 to assess the appropriate size distribution prior to microarray hybridization.

#### Target Hybridization, Washing, Staining and Scanning

GeneChip M430A probe arrays (Affymetrix) were hybridized, washed and stained according to the manufacturer's instructions in a fluidics station. The arrays were scanned using a Hewlett Packard confocal laser scanner and visualized using GeneChip 5.1 software. Three data files were created, namely image data (.dat), cell intensity data (.cel) and expression probe analysis data (.chp) files. Detailed protocols for sample preparation and target labeling assays for expression analysis can be found at http://www.Affymetrix.com.

### GeneChip Data Access

The entire microarray probe-set from each GeneChip 430A and individual CBA mouse phenotypic (hearing measures) data have been submitted to the GEO-NCBI database, and have been approved with the following Series reference #: GSE GSE49543. These GeneChip data can be accessed via, http://www.ncbi.nlm.nih.gov/geo/query/acc.cgi?acc=GSE49543. The probe-sets used in the current article were derived from the above GEO-NCBI accession number GSE49543. All steps were conducted according to the MIAME (Minimum Information About a Microarray Experiment) checklist [61].

### Real-time PCR (qPCR)

The primer/probe used in quantification of gene expression was acquired from TaqMan® Gene Expression Assays-on-Demand products (AOD) from Applied Biosystems, Inc. (Foster City, CA, USA).

Detailed information about the PCR protocols is found in www.appliedbiosystems.com, and in our previous microarray articles [26]–[28].

### Statistical Analyses

#### GeneChip Expression Analysis

After assessing chip quality, the Affymetrix GeneChip Operating Software (GCOS) automatically generates the (.cel) image file from the (.dat) data file. The signal log ratio of each sample determined the difference in expression of the studied gene in that sample from the mean expression of that gene in all samples from the young adult mice. A signal log ratio of 1.0 indicates an increase of the transcript level by 2 fold and −1.0 indicates a decrease by 2 fold. A signal log ratio of zero indicates no change.

For the fifty-six antioxidant-related genechip probes, one-way Analysis of Variance (ANOVA) (95% confidence limit) was used to compare between the signal log ratio values of the different subject groups. In addition, fold changes of all samples were calculated from signal log ratios using the following equations: 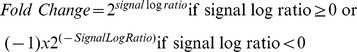



### Real Time PCR Analysis

The threshold cycle (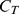) values were measured to detect the threshold of each of four significant genes of interest and *GAPDH* gene in all samples [32]–[33]. Each sample was measured in triplicate and normalized to the reference *GAPDH* gene expression. The 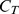 value of each well was determined and the average of the three wells of each sample was calculated. For samples that showed no expression of the test gene, the value of minimum expression was used for statistical analysis.

Delta 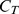
(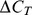) for test gene of each sample was calculated using the equation: 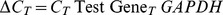



Delta delta 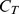
(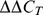) was calculated using the following equation: 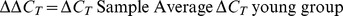



The fold change in the test gene expression was finally calculated from the formula: 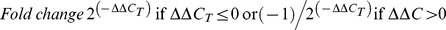



A statistical evaluation of real time PCR results was performed using one-way ANOVA to compare between 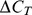 for test gene expression in young age, middle age, old age mild presbycusis, and old age severe presbycusis groups.

For the three significantly different genes on genechip and/or qPCR, linear regression analyses were employed to find correlations between the signal log ratio values or fold change and the functional hearing measurements. These measurements were the distortion product otoacoustic emission (DPOAE) amplitudes at low frequencies (5.6 kHz to 14.5 kHz), mid frequencies (15.8 kHz to 29.0 kHz) and high frequencies (31.6 kHz to 44.8 kHz), in addition to auditory brainstem response thresholds (ABR) at 3, 6, 12, 24, 32 and 48 kHz. The GraphPad Prism 4 software was used to perform the one-way ANOVA and the linear regression statistics.

## Results

### Subject Groups: Age and Hearing Functionality

The sample set segregated into 4 groups based upon age, DPOAE levels and ABR thresholds: young adult control with good hearing (N = 8, 4 males, 4 females, age = 3.5+/−0.4 mon), middle-aged with good hearing (N = 17, 8 males, 9 females, age = 12.3+/−1.3 mon), old with mild presbycusis (N = 9, 4 males, 5 females, age = 27.7+/−3.4 mon) and old with severe presbycusis (N = 6, 2 males, 4 females, age = 30.6+/−1.9 mon). Detailed audiological data were presented in Tadros et al. [26]–[27].

### Microarray gene expression

Fifty-six antioxidant-related gene probes were present on the microarray, and these were selected for further analysis. Statistical computations comparing the *signal log ratios* (Table 1) and *fold changes* of middle age, old mild hearing loss, and old severe hearing loss subject groups showed significant differences in expression or large fold-changes for four genes: *Gpx6, Txnrd1, Idh1*, and *Hspb1*. These gene expression changes were selected for validation by using real-time PCR (qPCR). In our selection of the genes to validate with real-time PCR, we relied on either a significant statistical difference of gene expression between groups (e.g., in the case of Txnrd1 and Idh1) or a large numerical difference in the microarray average fold change of the young adult group compared to the middle and old groups (e.g., in the case of Hspb1 and Gpx6). For Hspb1 and Gpx6, although the signal log ratio differences were not statistically significant, notice that there is a strong trend for gene expression group differences.

**Table 1:** ANOVA results of genechip signal log ratios between groups and average fold changes of validated genes.

Gene ID	Gene Name	ANOVA (Sig. Log. Ratio)	GeneChip Average Fold Change		
			Middle Age	Old Mild	Old Severe
**1425964_x_at**	Hspb1	P = 0.0598, F = 3.11, df = 2,29	−0.7729	−1.245	0.5188
**1452135_at**	Gpx6	P = 0.0681, F = 2.95, df = 2,29	0.9848	1.550	1.310
**1421529_a_at**	Txnrd1	P = *0.0219, F = 4.37, df = 2,29	−0.1404	−1.138	−1.485
**1419821_s_at**	Idh1 (NADP+)	P = *0.0131, F = 5.05, df = 2,29	−0.2129	−1.032	−1.612

^*^ denotes statistical significance at 0.05 level.

### Real-time PCR (qPCR)

**Figure 1:**
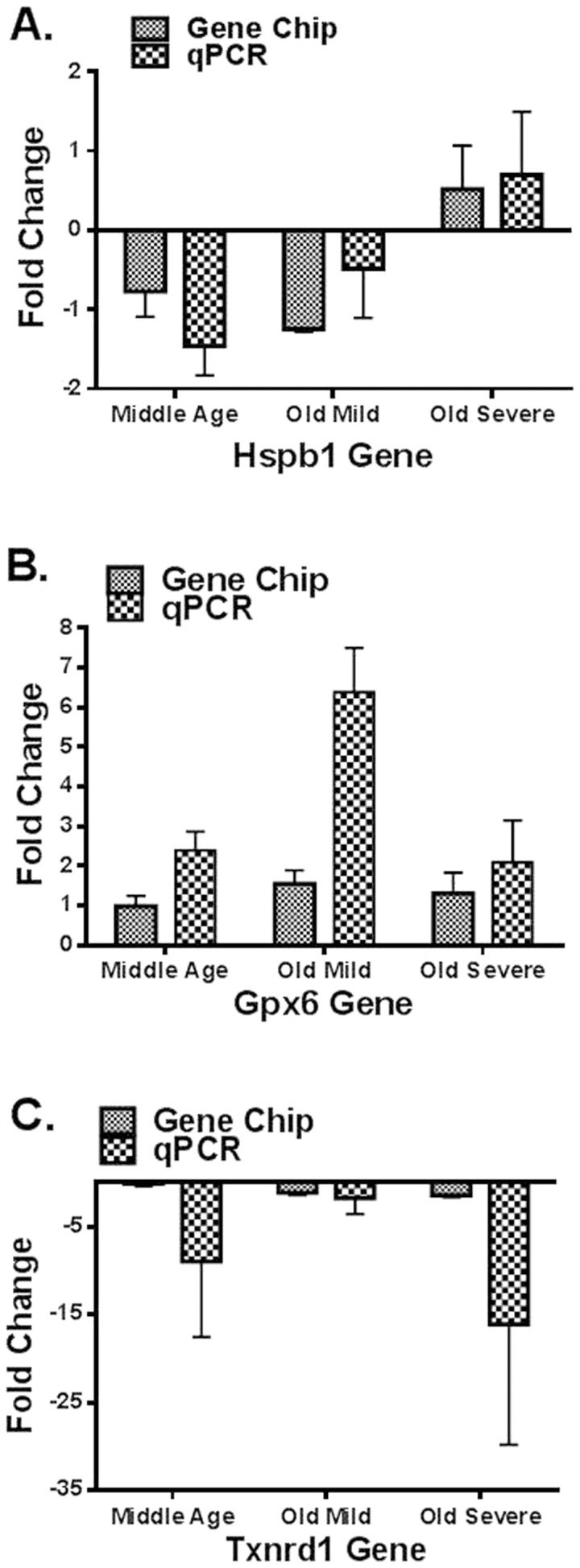
A): For both GeneChip and real-time PCR, fold changes of *Hspb1* gene expression in the cochleae of middle age, old mild hearing loss, and old severe hearing loss groups showed upregulation with age and hearing loss. B): For both GeneChip and real-time PCR, fold changes of *Gpx*6 gene expression in cochlea samples showed upregulation in all age groups compared to the young group. C): For both GeneChip and real-time PCR, fold changes of *Txnrd1* gene expression in the cochleae of middle age, old mild hearing loss, and old severe hearing loss groups showed downregulation with age and hearing loss.

Three genes out of the four validated microarray genes showed comparable results for both the genechip and the qPCR. These three genes were: *Hspb1* (p = *0.0345, F = 3.79, df = 2,29) and *Gpx6* (p = **0.0011, F = 8.644, df = 2,29) that showed significant up-regulation and *Txnrd1* (p = 0.6575, F = 0.4254, df = 2,29) that showed down-regulation with age and hearing loss. For *Idh1* gene, the qPCR results did not show consistency with the genechip data. Figures 1A–1C show quantitative comparisons between fold changes for the genechip microarray expression changes and those for the qPCR.

### Correlation between gene expression and hearing measurements

**Figure 2:**
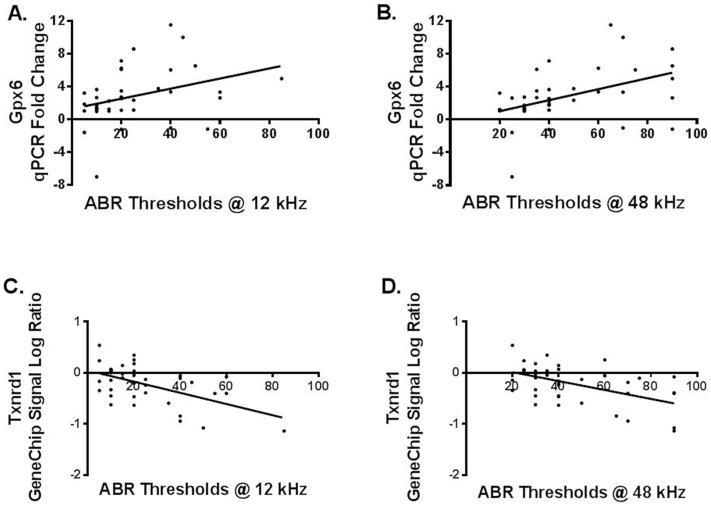
ABR thresholds correlation with gene expression A) and B): The correlations between *Gxp6* qPCR fold changes and ABR thresholds at 12 kHz and 48 kHz are two examples of the significant correlations of gene expression changes with ABR test results. C) and D): The correlations between *Txnrd1* signal log ratio and ABR thresholds at 12 kHz and 48 kHz were significant.

**Figure 3:**
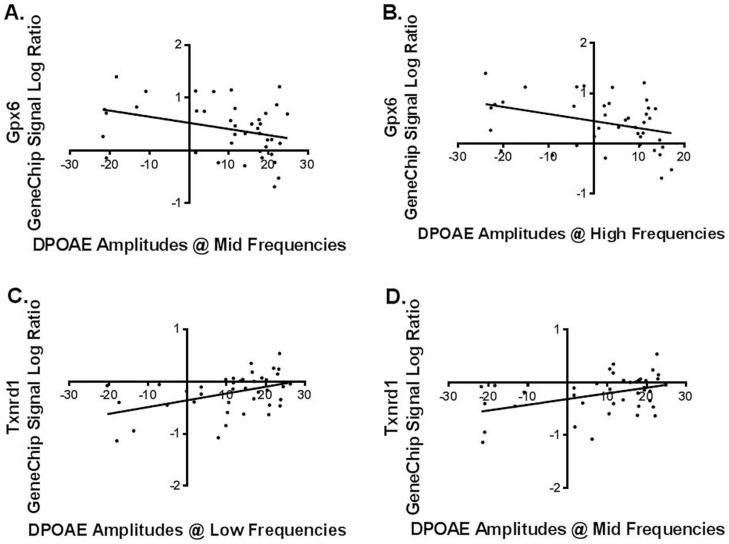
DPOAE amplitudes correlation with gene expression A) and B): The correlations between *Gxp6* signal log ratio and DPOAE amplitudes at mid and high frequencies, are two examples of the significant correlations of gene expression changes with DPOAE test results. C) and D): The correlations between *Txnrd1* signal log ratio and DPOAE amplitudes at low and mid frequencies were significant.

Linear regression tests were used to analyze the correlations between hearing measurements (ABR thresholds and DPOAE amplitudes) and gene expression for both the genechip and qPCR expression levels. For *Gpx6* expression upregulation, genechip and q-PCR data showed significant correlations with both ABR thresholds and DPOAE amplitudes (Table 2) (Figs. 2A–B, 3A–B). For *Txnrd1* gene expression down-regulation, genechip signal log ratios and fold changes showed statistically significant correlations with ABR thresholds and DPOAE amplitudes (Table 3) (Fig. 2C–D, 3C–D). No significant correlations were found between *Hspb1* gene expressions as measured by qPCR and audiological tests. This may be, because the qPCR method is overall more sensitive than the genechip, for individual gene analyses. When the qPCR results are used to calculate fold changes, the results can have more variability than the genechip fold changes, sometimes reducing the statistical power of the correlations.

**Table 2:** Correlation between Gpx6 gene expression and audiological measurements.

	Sig. Log Ratio (GeneChip)	Fold Change (GeneChip)	Fold Change (qPCR)
**ABR 3 kHz**	p = 0.187, r^2^ = 0.045, F = 1.803	p = 0.239, r^2^ = 0.0362, F = 1.426	p = 0.186, r^2^ = 0.046, F = 1.815
**ABR 6 kHz**	p = 0.065, r^2^ = 0.087, F = 3.618	p = 0.113, r^2^ = 0.0648, F = 2.632	p = 0.075, r^2^ = 0.081, F = 3.352
**ABR 12 kHz**	p = * **0.019**, r^2^ = 0.137, F = 6.028	p = * **0.047**, r^2^ = 0.100, F = 4.237	p = * **0.029**, r^2^ = 0.119, F = 5.170
**ABR 24 kHz**	p = * **0.014**, r^2^ = 0.149, F = 6.658	p = 0.051, r^2^ = 0.0963, F = 4.050	p = * **0.048**, r^2^ = 0.0989, F = 4.175
**ABR 32 kHz**	p = * **0.019**, r^2^ = 0.136, F = 5.984	p = 0.052, r^2^ = 0.0959, F = 4.031	p = * **0.022**, r^2^ = 0.130, F = 5.699
**ABR 48 kHz**	p = ** **0.002**, r^2^ = 0.222, F = 10.84	p = * **0.011**, r^2^ = 0.159, F = 7.186	p = ** **0.004**, r^2^ = 0.204, F = 9.721
**DPOAE Low Freq.**	p = 0.117, r^2^ = 0.0634, F = 2.572	p = 0.269, r^2^ = 0.0319, F = 1.255	p = 0.579, r^2^ = 0.008, F = 0.312
**DPOAE Mid Freq.**	p = * **0.037**, r^2^ = 0.109, F = 4.652	p = 0.159, r^2^ = 0.0515, F = 2.064	p = 0.237, r^2^ = 0.037, F = 1.44
**DPOAE High Freq.**	p = * **0.027**, r^2^ = 0.123, F = 5.305	p = 0.119, r^2^ = 0.0625, F = 2.535	p = 0.217, r^2^ = 0.039, F = 1.576

^*^ denotes statistical significance at 0.05 level;

^**^ at the 0.01 level.

**Table 3:** Correlation between *Txnrd1* gene expression and audiological measurements.

	Sig. Log Ratio (GeneChip)	Fold Change (GeneChip)	Fold Change (qPCR)
**ABR 3 kHz**	p = 0.083, r^2^ = 0.0768, F = 3.161	p = 0.077, r^2^ = 0.079, F = 3.304	p = 0.516, r^2^ = 0.011, F = 0.429
**ABR 6 kHz**	P = ** **0.004**, r^2^ = 0.195, F = 9.189	p = ** **0.006**, r^2^ = 0.180, F = 8.339	p = 0.561, r^2^ = 0.009, F = 0.345
**ABR 12 kHz**	p = *** **0.0003**, r^2^ = 0.293, F = 15.75	p = *** **0.0009**, r^2^ = 0.255, F = 13.00	p = 0.833, r^2^ = 0.0012, F = 0.045
**ABR 24 kHz**	p = ** **0.002**, r^2^ = 0.222, F = 10.87	p = ** **0.006**, r^2^ = 0.181, F = 8.395	p = 0.849, r^2^ = 0.0009, 0.037
**ABR 32 kHz**	p = ** **0.0066**, r^2^ = 0.179, F = 8.272	p = ** **0.0079**, r^2^ = 0.172, F = 7.875	p = 0.746, r^2^ = 0.0028, F = 0.107
**ABR 48 kHz**	p = *** **0.0006**, r^2^ = 0.269, F = 13.96	p = ** **0.0012**, r^2^ = 0.245, F = 12.36	p = 0.909, r^2^ = 0.0003, F = 0.013
**DPOAE Low Freq.**	p = ** **0.005**, r^2^ = 0.188, F = 8.822	p = ** **0.0065**, r^2^ = 0.179, F = 8.283	p = 0.355, r^2^ = 0.0226, F = 0.877
**DPOAE Mid Freq.**	p = ** **0.008**, r^2^ = 0.171, F = 7.818	p = ** **0.004**, r^2^ = 0.195, F = 9.213	p = 0.478, r^2^ = 0.013, F = 0.515
**DPOAE High Freq.**	p = * **0.034**, r^2^ = 0.113, F = 4.823	p = ** **0.0075**, r^2^ = 0.174, F = 7.981	p = 0.598, r^2^ = 0.007, F = 0.282

^*^ denotes statistical significance at 0.05 level;

^**^ at the 0.01 level;

^***^ at the 0.001 level.

## Discussion

### Antioxidant Systems

The antioxidant systems, thiol-reducing Glutathione (GSH) and thioredoxin (Trx), are the main cellular defense mechanisms against free radicals. Catalyzed by the glutathione and thioredoxin peroxidases, GSH and Trx can scavenge free radicals and reduce peroxides producing glutathione disulfide (GSSG) and thioredoxin disulfide, respectively. This oxidized form of glutathione and thioredoxine can be converted back to the reduced form by glutathione and thioredoxine reductases in a reaction requiring NADPH. Since glutathione is the most abundant intracellular redox, in addition to its major role as an antioxidant, the GSH/GSSG ratio is considered as an indicator of the cellular redox environment and has a major influence on cell proliferation and differentiation [20], [34]. Thioredoxin system has an equally important role as an antioxidant and redox regulating system. In addition it plays a key role in DNA synthesis and activation of transcription factors that regulate cell growth [21]. In cases of oxidative stress, these two antioxidant systems act, both in parallel and interactive ways, to reduce ROS/RNS concentration and regulate redox cellular environment [17], [35].

Glutathione peroxidase (Gpx) isoforms are a family of enzymes that slightly vary in properties [36]. The classic intracellular *Gpx1* is expressed in varies types of tissues and *Gpx3* is found intensively in plasma, kidney, adrenal gland, and weakly in heart, lung and cerebellum [37]–[38]. *Gpx6* has been identified based on its close homology with *Gpx3* [39] and expressed in embryos and adult olfactory epithelium. The expression of Gpx isoforms differs depending on tissue type.

Thioredoxin reductase (Txnrd) is a selenoprotein. It has three isoforms *Txnrd1* (cytosolic), *Txnrd2* (mitochondrial) and *Txnrd3* (TGR or thioredoxin and glutathione reductase) [40]–[45]. The inhibition of thioredoxine reductase enzyme may have a devastating effect on the cell due to the inhibition of the whole thioredoxin system [46]. In addition to its antioxidant effect, it has been reported that *Txnrd1* plays an important role in the control of basal p53 activity [47].

Heat shock proteins (Hsps) are a multi-functional family of proteins. Under stressful conditions, as infection, inflammation, exposure to toxins, elevated temperature or other stress conditions, Hsps have a cytoprotective function through working as a molecular chaperon to repair or remove denatured proteins and inhibit apoptosis [48]–[49].

### Antioxidants and Cochlea

Different tissues in the cochlea may respond differently to the damaging ROS but the effects of oxidative stress and antioxidant systems on aging cochlea are well known [50]–[52]. Some reports showed a significant lower activity of glutathione-related antioxidant enzymes with noise exposure and drugs [53]–[55]. Mutation of the gene of *Gpx1* was reported to increase the vulnerability to noise-induced and age-related hearing loss in mice [56]. Ebselen, a Gpx mimic seleno-organic compound, was found to have a protective effect on cochlea against acoustic trauma, gentamicin and cisplatin [57]–[58]. Though the role of thioredoxin system in the central nervous system has been established [16], its protective effect in the cochlea is still needed to be explored. It was reported that *Txnrd* activation in strial melanocytes by low level acoustic stimulation (LLAS) might be the cause of reduced susceptibility to temporary noise-induced threshold shift [59]. *Hsps* are divided into families according to their molecular weight. Heat shock protein b1 (*Hspb1*), also called Hsp27, has been found in the outer hair cells of rat cochlea and may provide a protection to these cells against auditory trauma or oxidative stress. In addition, the presence of high levels of Hsp27 in the cuticular plate and lateral wall of outer hair cells may help the regulation of the actin cytoskeleton against mechanical strain [60]. A limitation of the present study is that we cannot be sure about the importance of the up-regulation of Hspb1 expression in old age with severe hearing loss. It could be explained as an attempt to protect the cochlear hair cells from age-linked acoustic trauma and stress, which requires further investigation.

In the present investigation, *Txnrd1* was found to be downregulated while both *Gpx6* and *Hspb1* were upregulated in the CBA mouse cochlea with age and hearing loss. These findings may be explained as an exhaustion of the thioredoxin system with oxidative stress, and a compensatory mechanism by both the glutathione system and heat shock proteins in cochlear cells to scavenge the excess free radicals. *Hspb1* changes showed upregulation only in the old severe hearing loss group that may indicate its main relation with hearing loss. Though further studies may be needed to provide the functional and histological proof of the protective effect of antioxidants on age related hearing loss, the strong correlations between both *Txnrd1* or *Gpx6* expression on one hand and ABR thresholds and DPOAE amplitudes on the other hand, point to direct relations of the expression of these genes in the cochlea with age and hearing loss. The downregulation of *Txnrd1* expression may have a special therapeutic significance for future efforts at prevention and/or treatment of presbycusis through gene therapy techniques.

Recently, Tanaka et al. [62] studied the expression pattern of 84 oxidative stress and antioxidant defense-related genes in F344/NHsd male albino rates, dividing their subject groups *solely* according to age. In the present study, we used CBA/CaJ mice, both males and females, to study 56 oxidative stress-related genes and divided the animals into four groups according to two criteria, *both* their age and audiological test (ABR and DPOAEs) results. This categorization helped us to more clearly differentiate between the effects of age and degree of hearing loss in the old age groups, on the oxidative stress gene expression. Tanaka and colleagues reported a statistically significant difference in expression between age groups in thirteen genes (one was down-regulated and twelve were up-regulated with age). Five genes were glutathione- and thioredoxin-related (Gpx6, Gpx3, Gstk1, Txnip, and Gsr). The expression of these genes was correlated to age but not to hearing loss. Note that Tanaka et al. grouped their data for multiple sound frequencies, whereas correlations were made with individual sound frequencies in the present study. Many of the genes that were correlated to both age and hearing loss have cytoprotective functions or have both survival and apoptotic function (Scd1, Cygb, Duo2, Aass, Slc38a5, Nqo1, Vim, and Dhcr24). In terms of similarities, Tanaka et al. reported changes in the Txnip gene, and we observed the down-regulation of a related gene: thioredoxin reductase 1 (Txnrd1). Note that Tanaka et al. did not find a correlation between the expression of Gpx6 and the degree of hearing loss, but both studies confirm the importance of this gene that is upregulated with age in both the mouse and rat cochlea, presumably to help preserve hearing in old age. The differences in findings for other genes may relate to the different species employed, the type of microarray utilized, or differences in the analysis of the microarray and qPCR results carried out in both studies. Lastly, notice also that the individual effects of age and of hearing loss in the expression patterns could not be clearly addressed in Tanaka et al.'s study, and this issue will be interesting to address in subsequent investigations.
